# Long-Term Effect on Health-Related Quality of Life in Patients With COVID-19 Requiring Hospitalization Compared to Non-hospitalized COVID-19 Patients and Healthy Controls

**DOI:** 10.7759/cureus.31342

**Published:** 2022-11-10

**Authors:** Emmanouil Koullias, Georgios Fragkiadakis, Maria Papavdi, Georgia Manousopoulou, Triantafyllia Karamani, Helena Avgoustou, Evangelia Kotsi, Dimitris Niakas, Dimitrios Vassilopoulos

**Affiliations:** 1 2nd Department of Internal Medicine, Hippocration General Hospital, National and Kapodistrian University of Athens, Athens, GRC; 2 School of Social Sciences, Hellenic Open University, Patra, GRC; 3 School of Health Sciences, Department of Health Economics, National and Kapodistrian University of Athens, Athens, GRC

**Keywords:** post-covid-19, eq5d5l, facit-f, health-related quality of life, sf36v2, severe covid-19

## Abstract

Background

In this study, we aimed to evaluate the health-related quality of life (HRQL) in patients with severe coronavirus disease 2019 (COVID-19) six months after their hospitalization and compare it to that of non-hospitalized patients with mild COVID-19 and healthy controls.

Methodology

Participants were enrolled between September 2021 and April 2022 and included hospitalized COVID-19 patients at General Hospital of Athens “Hippocration” who had been discharged at least six months prior to enrollment, non-hospitalized patients with COVID-19, and healthy controls. Collected data included demographics, disease severity, medication history, and comorbidities. Participants completed a EuroQol 5 Dimensions 5Levels (EQ5D5L), a Short Form 36 version 2 (SF36v2), a Functional Assessment of Chronic Illness Therapy-Fatigue (FACIT-F), and a Post-COVID-19 Functional Status Scale (PCFSS) regarding HRQL before and six months after infection with severe acute respiratory syndrome coronavirus 2. In the case of healthy controls, two sets of questionnaires were completed at least six months apart. Statistical analysis was performed using the SPSS version 25 software (IBM Corp., Armonk, NY, USA).

Results

A total of 151 participants were enrolled. Hospitalized patients with COVID-19 demonstrated a statistically significant deterioration in most parameters of SF36v2 as well as both parameters of the EQ5D5L and FACIT-F questionnaires. Hospitalized patients exhibited worse results in SF36v2 and EQ5D5L when compared to both healthy controls as well as those with mild COVID-19 (p < 0.05). Hospitalized women, in particular, were shown to fare worse than other women in parameters associated with both mental/psychological and physical health (p < 0.05). Hospitalized patients between 41 and 60 years old demonstrated a statistically significant drop in the scores of all three main questionnaires compared to their previous health status (p < 0.05). Hospitalized patients between 61 and 80 years old exhibited a similar trend, but statistical significance was achieved in fewer parameters. HRQL decline was greater in both age groups compared to that of healthy and milder disease counterparts. There was a significant correlation between the results from the three main questionnaires. Similarly, PCFS scale values were shown to correlate with disease severity (hospitalization or not) and age.

Conclusions

HRQL remained noticeably impacted six months after hospitalization due to COVID-19. The physical and mental/psychological stress of severe COVID-19 translated into lasting health deterioration, especially for women and those aged 41-60 years old. The use of questionnaires, such as those implemented in this study, might help in the early detection of patients who could benefit from rehabilitation programs. Psychological, as well as physical and social, support is crucial to alleviate the burden of post-COVID-19 symptomatology and expedite the recovery of this group of patients.

## Introduction

The current pandemic caused by the severe acute respiratory syndrome coronavirus 2 (SARS-CoV-2) has been the root of severe damage to social, health-related policy, and economic norms globally. The millions of deaths are accentuated by a much larger number of long-term affected survivors. The resulting decline in health-related quality of life (HRQL) after hospitalization due to coronavirus disease 2019 (COVID-19) has been shown to persist for various time periods after discharge in several studies globally [1-6] and is expressed through a rather diverse symptomatology encompassing practically all systems, collectively labeled as post-COVID-19 syndrome [7-13]. The exact duration of such symptoms has not yet been defined, while groups such as women, the elderly, those with comorbidities, those belonging to a lower socioeconomic class, and those who experienced a severe course of the disease appear to be in greater danger to develop such symptoms [14]. Although no particular treatment is available, programs of physical rehabilitation and psychological support appear to help those who undergo an arduous recovery [15-19].

Our objective was to evaluate the long-term effect (six months) on HRQL following severe COVID-19 requiring hospitalization, compared to mild COVID-19 not requiring hospitalization, through the use of several different questionnaires and scales. Furthermore, trends indicating certain groups of patients susceptible to long COVID-19 were investigated, while at the same time possible correlations between different questionnaires’ results were explored. These scales could be used in clinical settings to detect patients who could probably benefit from rehabilitation.

## Materials and methods

Study design and participants

This was a single-center study that included patients with severe COVID-19 who were hospitalized in the COVID-19 Unit of the General Hospital of Athens “Hippocration” while non-hospitalized patients with COVID-19 and healthy individuals served as controls. COVID-19 was diagnosed with a positive polymerase chain reaction (PCR) test in all cases. Severe COVID-19 was specified as hypoxemia due to SARS-CoV-2 pneumonia which warranted hospitalization. The enrollment took place between September 2021 and April 2022. At least six months had elapsed since the participants’ affliction with SARS-CoV-2 in the cases of mild and severe disease groups. Collected data from patients and controls included age, weight, height, and medical history. Subjects were given copies of a EuroQol 5 Dimensions 5 Levels (EQ5D5L), a Short Form 36 version 2 (SF36v2), a Functional Assessment of Chronic Illness Therapy-Fatigue (FACIT-F), and a Post-COVID-19 Functional Status Scale (PCFSS) questionnaires, translated into the Greek language, and were asked to complete them taking into regard their health condition as it was before the disease and at the time of participation. From the EQ5D5L questionnaire, the parameters EuroQol Visual Analogue Scale (EQ VAS) and the Index Value, while from the SF36v2 the parameters Physical Function (PF), Role Physical (RP), Body Pain (BP), General Health (GH), Vitality (VT), Social Functioning (SF), Role Emotional (RE), and Mental Health (MH) were calculated. From the eight subscores of SF36v2, the Physical Health Score (PHS) and the Mental Health Score (MHS) were determined. Higher scores in these scales indicated better performance, with the exception of PCFSS, where higher scores corresponded with a greater degree of disability.

SF36v2 is a widely used scale incorporated in a large spectrum of research and one of the first to be utilized for COVID-19-related studies [14]. Similarly, EQ5D5L and FACIT-F have been applied in various studies internationally regarding the effects of the COVID-19 pandemic, among others [20-23]. Additionally, PCFSS is a recently created tool, aiming for quick assessment of possible long-term loss of function resulting from a SARS-CoV-2 infection [24,25]. The SF36v2, EQ5D5L, and FACIT-F were already validated and available in the Greek language [26-30], while the PCFSS was translated for the needs of the study.

Statistical analysis

Descriptive statistics were calculated. Comparisons between subgroups were performed using the t-test for normally distributed data, while the Wilcoxon signed ranks test was employed for non-normally distributed data. The prespecified threshold for statistical significance was set at <0.05. Analyses were conducted using SPSS version 25 software (IBM Corp., Armonk, NY, USA).

## Results

Patient and control characteristics

A total of 151 participants were enrolled in the study; 88 with COVID-19 (37 hospitalized, 51 non-hospitalized, comprising the severe COVID-19 group) and 63 healthy controls. Of the total, 78 were men, and 73 were women. The milder course participants (26 men and 25 women, comprising the mild COVID-19 group) as well as healthy controls (27 men and 36 women) were balanced regarding the two sexes. As far as the composition of the severe COVID-19 group is concerned, men were affected more often than women (68% vs. 32%). The mean age of men was 48.99 (SD = 16.46) years and of women was 43.45 (SD = 15.01) years.

Hospitalized patients were older (mean age = 61.86 ± 8.41 years) compared to non-hospitalized patients (mean age = 39.49 ± 13.97 years) and healthy controls (mean age = 42.70 ± 14.92 years). This was the case both for men (63.92 ± 8.53 vs. 39.69 ± 14.50 vs. 44.11 ± 14.41 years) and women (57.58 ± 6.59 vs. 39.28 ± 13.69 vs. 41.64 ± 15.40 years).

The mean body mass index (BMI) was 28.87 kg/m^2^ (SD = 4.91) for men and 25.62 kg/m^2^ (SD = 4.69) for women. Women in the severe COVID-19 group had a statistically significant greater weight and BMI than women in the mild COVID-19 group. Baseline characteristics are shown in Table 1. Optimal treatment and support were offered during the hospital stay, according to the updated national guidelines at the time.

**Table 1:** Baseline characteristics of the study sample. *Women of SC had statistically significantly greater weight and BMI than women of MC. **Both men and women of SC were significantly older than those of MC, H. BMI: body mass index; CKD: chronic kidney disease; COPD: chronic obstructive pulmonary disease; COVID-19: coronavirus disease 2019; DM: diabetes mellitus; HC: healthy controls; HF: heart failure; M: men; MC: mild COVID-19; SC: severe COVID-19; SD: standard deviation; W: women

Characteristics		Value	Percentage (%)
Sex	Men	78	51.7
	Women	73	48.3
Group	Severe COVID-19	37	24.5
	Mild COVID-19	51	33.8
	Healthy controls	63	41.7
Bodyweight, kg; mean (SD)	Men	90.51 (15.97)	
	SC-MC-HC	90.16-90.46-90.89; p = 0.920	
	Women	68.27 (13.73)	
	SC-MC-HC	79.46-63.42-67.90; p = 0.022*	
Height, m; mean (SD)	Men	1.77 (0.06)	
	SC-MC-HC	1.74-1.81-1.75	
	Women	1.63 (0.06)	
	SC-MC-HC	1.63-1.63-1.63	
BMI, kg/m^2^; mean (SD)	Men	28.87 (4.91)	
	SC-MC-HC	29.57-27.48-29.56	
	Women	25.62 (4.69)	
	SC-MC-HC	29.68-23.96-25.41; p = 0.009*	
Age, years; mean (SD)	Men	48.99 (16.46)	
	SC-MC-HC	63.92-39.69-44.11; p = 0.000**	
	Women	43.45 (15.01)	
	SC-MC-HC	57.58-39.28-41.64; p = 0.002**	
Presence of comorbidities (DM, hypertension, HF, COPD, obesity, CKD, immunosuppression, smoking)	Severe COVID-19 (M-W)	18/25-10/12	72.0-83.3
	Mild COVID-19 (M-W)	9/26-5/25	34.6-20.0
	Healthy controls (M-W)	13/27-9/36	48.1-25.0

Analysis

Hospitalized patients with COVID-19 exhibited statistically significant deterioration in the FACIT-F Fatigue questionnaire (p = 0.015) six months after discharge. Furthermore, this group had a notable decline in several parameters of the SF36v2, such as PF (p = 0.005), RP (p = 0.021), GH (p = 0.005), VT (p = 0.0015), SF (p = 0.040), and MH (p = 0.003) compared to their former performance. These observations were also reflected in a statistically significant drop as far as the composite PHS (p = 0.034) and MHS (p = 0.004) were concerned. Regarding the EQ5D5L, both EQ VAS (p = 0.004) and the Index Value (p = 0.006) were impacted in hospitalized patients (Table 2).

**Table 2:** Comparison of SF36v2, EQ5D5L, and FACIT-F outcomes of severe COVID-19 group between baseline (before infection) and at least six months after severe COVID-19. BP: Body Pain; COVID-19: coronavirus disease 2019; EQ5D5L: EuroQol 5 Dimensions 5 Levels; EQ VAS: EuroQol Visual Analogue Scale; FACIT-F: Functional Assessment of Chronic Illness Therapy-Fatigue; GH: General Health; MH: Mental Health; MHS: Mental Health Score; PF: Physical Function; PHS: Physical Health Score; RE: Role Emotional; RP: Role Physical; SD: Standard Deviation; SF36v2: Short Form 36 version 2; SF: Social Functioning; VT: Vitality

Questionnaire	Mean (SD) at baseline	Mean (SD) ≥6 months later	P-value
SF36v2			
PF	82.16 (18.95)	73.65 (21.66)	0.005
RP	84.63 (21.28)	76.18 (27.83)	0.021
BP	76.70 (22.39)	70.62 (28.27)	0.204
GH	67.81 (20.53)	60.65 (22.15)	0.005
VT	66.35 (14.37)	56.76 (20.79)	0.0015
SF	82.24 (22.26)	75.84 (26.34)	0.040
RE	84.43 (19.89)	78.59 (23.68)	0.078
MH	64.86 (17.03)	60.00 (20.61)	0.003
MHS	52.52 (6.22)	49.25 (9.73)	0.004
PHS	41.30 (11.13)	37.76 (13.12)	0.034
FACIT-F			
	40.89 (9.27)	37.89 (11.54)	0.015
EQ5D5L			
EQ VAS	79.41 (15.44)	73.78 (17.65)	0.004
Index Value	0.855 (0.189)	0.788 (0.244)	0.006

On the other hand, the mild COVID-19 group exhibited a decrease in VT (p = 0.035) from the SF36v2 and a similar tendency of the Index Value from the EQ5D5L, while healthy controls exhibited an improvement in the parameters RP (p = 0.005) and PHS (p = 0.031).

The PCFS scale generally had higher values for the severe COVID-19 group compared with the mild COVID-19 group, and the chi-square test showed a statistically significant correlation between disease severity and loss of function in everyday life activities (p = 0.036, Cramer’s V = 0.342).

Subgroup analysis based on sex (Tables 3, 4) showed that hospitalized women exhibited a significant drop in the FACIT-F score (p = 0.022) as well as several parameters of the SF36v2 questionnaire. Specifically, PF was impacted (p = 0.0215), along with RP (p = 0.048), GH (p = 0.017), VT (p = 0.022), MH (p = 0.0145), and the aggregates PHS (p = 0.0345) and MHS (p = 0.033). On the other hand, healthy women exhibited an improvement of RP (p = 0.002) and PHS (p = 0.0195) and a deterioration of SF (p = 0.028), while women in the mild COVID-19 group had a drop in VT (p = 0.0275) as the only significant finding. Men in the severe COVID-19 group had a decline regarding VT (p = 0.0145), MH (p = 0.04), and MHS (p = 0.018), while healthy men had no significant findings compared to their past, and men in the mild COVID-19 group had only a drop in SF (p = 0.036). Similarly, as far as the EQ5D5L is concerned, women in the severe COVID-19 group exhibited deterioration regarding both the EQ VAS (p = 0.000) and the Index Value (p = 0.010), and women in the mild COVID-19 group showed deterioration of the Index Value (p = 0.028) only, while healthy women had no statistical difference. At the same time, men in all groups exhibited no statistically significant change, with the severe COVID-19 group only showing a tendency toward deterioration.

**Table 3:** Comparison of SF36v2 outcomes between severe COVID-19 group and mild/healthy cases based on sex. BP: Body Pain; COVID-19: coronavirus disease 2019; GH: General Health; IQR: interquartile range; MH: Mental Health; MHS: Mental Health Score; m/h: mild/healthy; PF: Physical Function; PHS: Physical Health Score; RE: Role Emotional; RP: Role Physical; SC: severe COVID-19 Group; SD: standard deviation; SF36v2: Short Form 36 version 2; SF: Social Functioning; VT: Vitality; Δ: change

SF36v2	Severe COVID-19 group (SC) vs mild/healthy (m/h) cases	Mean (SD)	Median (IQR)	P-value (comparison between present and past)	P-value (comparison between SC and m/h)
ΔPF	SC men	-5.00 (15.28)	0.00 (15.00)	0.065	0.026
	m/h	-1.79 (15.91)	0.00 (0.00)	0.475	
	SC women	-15.83 (23.92)	-10.00 (27.50)	0.0215	0.002
	m/h	1.64 (11.50)	0.00 (5.00)	0.155	
ΔRP	SC men	-4.50 (15.78)	0.00 (6.25)	0.084	0.068
	m/h	0.94 (18.16)	0.00 (0.00)	0.935	
	SC women	-16.67 (31.68)	-3.13 (40.63)	0.048	0.051
	m/h	5.12 (18.02)	0.00 (12.50)	0.024	
ΔBP	SC men	-1.44 (24.73)	0.00 (6.00)	0.789	0.895
	m/h	1.81 (15.51)	0.00 (0.00)	0.756	
	SC women	-15.75 (34.63)	-11.00 (36.75)	0.097	0.023
	m/h	-0.03 (15.38)	0.00 (0.00)	0.949	
ΔGH	SC men	-2.88 (8.97)	0.00 (8.50)	0.122	0.057
	m/h	1.45 (12.30)	0.00 (5.00)	0.394	
	SC women	-16.08 (23.07)	-7.50 (21.50)	0.017	0.000
	m/h	1.92 (11.33)	0.00 (5.00)	0.152	
ΔVT	SC men	-6.00 (12.91)	0.00 (15.00)	0.0145	0.194
	m/h	-1.42 (11.90)	0.00 (5.00)	0.101	
	SC women	-17.08 (25.98)	-17.50 (43.75)	0.022	0.050
	m/h	-3.61 (11.94)	0.00 (10.00)	0.024	
ΔSF	SC men	-4.48 (15.74)	0.00 (12.00)	0.224	0.013
	m/h	5.41 (16.72)	0.00 (12.00)	0.015	
	SC women	-10.42 (23.04)	-6.00 (12.75)	0.073	0.513
	m/h	-5.07 (16.87)	0.00 (12.00)	0.011	
ΔRE	SC men	-3.04 (10.74)	0.00 (8.00)	0.164	0.647
	m/h	-0.19 (21.58)	0.00 (8.00)	0.616	
	SC women	-11.67 (27.95)	-4.00 (37.75)	0.088	0.181
	m/h	0.54 (17.57)	0.00 (12.00)	0.947	
ΔMH	SC men	-3.84 (10.49)	0.00 (6.00)	0.040	0.017
	m/h	1.74 (11.46)	0.00 (4.00)	0.484	
	SC women	-7.00 (9.67)	-2.00 (15.00)	0.0145	0.052
	m/h	-1.64 (10.54)	0.00 (8.00)	0.229	
ΔMHS	SC men	-2.72 (6.13)	-0.61 (4.09)	0.018	0.096
	m/h	0.89 (9.51)	0.00 (4.09)	0.788	
	SC women	-5.23 (8.87)	-2.95 (15.06)	0.033	0.283
	m/h	-1.94 (8.58)	-0.50 (8.05)	0.041	
ΔPHS	SC men	-1.33 (5.95)	0.00 (4.98)	0.277	0.154
	m/h	0.017 (5.05)	0.00 (1.98)	0.657	
	SC women	-7.32 (12.57)	-3.65 (17.24)	0.0345	0.009
	m/h	1.30 (5.41)	0.00 (6.53)	0.0325	

**Table 4:** Comparison of EQ5D5L and FACIT-F outcomes between severe COVID-19 group and mild/healthy cases based on sex. COVID-19: coronavirus disease 2019; EQ5D5L: EuroQol 5 Dimensions 5 Levels; EQ VAS: EuroQol Visual Analogue Scale; FACIT-F: Functional Assessment of Chronic Illness Therapy-Fatigue; IQR: interquartile range; m/h: mild/healthy; SC: severe COVID-19 group; SD: standard deviation; Δ: change

EQ5D5L	Severe COVID-19 group (SC) vs mild/healthy (m/h) cases	Mean (SD)	Median (IQR)	P-vale (comparison between present and past)	P-value (comparison between SC and m/h)
ΔEQ VAS	SC men	-1.40 (10.50)	0.00 (6.50)	0.194	0.046
	m/h	1.58 (12.47)	0.00 (0.00)	0.832	
	SC women	-14.42 (10.11)	-14.00 (13.75)	0.000	0.000
	m/h	0.05 (8.07)	0.00 (0.00)	0.896	
ΔIndex Value	SC men	-0.030 (0.114)	0.000 (0.082)	0.133	0.104
	m/h	0.005 (0.114)	0.000 (0.000)	0.468	
	SC women	-0.143 (0.181)	-0.074 (0.321)	0.010	0.006
	m/h	-0.017 (0.117)	0.000 (0.009)	0.073	
ΔFACIT-F	SC men	-0.88 (5.89)	0.00 (3.00)	0.478	0.701
	m/h	-0.45 (7.47)	0.00 (2.00)	0.389	
	SC women	-7.42 (11.29)	-2.50 (14.50)	0.022	0.049
	m/h	-0.46 (7.61)	0.00 (5.00)	0.433	

Subgroup analysis based on age (Tables 5, 6) showed that those aged 41 to 60 in the severe COVID-19 group exhibited a substantial decline in the FACIT-F score (p = 0.0325) and most parameters of the SF36v2 including PF (p = 0.004), BP (p = 0.01), GH (p = 0.01), VT (p = 0.037), SF (p = 0.0265), MH (p = 0.0095), MHS (p = 0.0105), and PHS (p = 0.024), as well as both parameters of the EQ5D5L, including EQ VAS (p = 0.005) and Index Value (p = 0.036). On the same note, those aged 61 to 80 in that group exhibited notable deterioration in RP (p = 0.024), VT (p = 0.0265), and MHS (p = 0.025) from the SF36v2 and in the Index Value (p = 0.04) from the EQ5D5L six months after hospitalization. On the contrary, all age subgroups from both healthy individuals and those with a mild infection from SARS-CoV-2 in their past exhibited no decline in the FACIT-F and EQ5D5L and most parameters of the SF36v2. The only findings were a decline in VT (p = 0.023), MH (p = 0.0475), and MHS (p = 0.0475) for the healthy-31 to 40, as well as a decline in VT of the mild COVID-19 group-18 to 30 (p = 0.0435) and mild COVID-19 group-31 to 40 (p = 0.0235). The healthy-41 to 60 exhibited improvement in RP (p = 0.008).

**Table 5:** Comparison of SF36v2 outcomes between the severe COVID-19 group and mild/healthy cases based on age. BP: Body Pain; COVID-19: coronavirus disease 2019; GH: General Health; IQR: interquartile range; MH: Mental Health; MHS: Mental Health Score; m/h: mild/healthy; PF: Physical Function; PHS: Physical Health Score; RE: Role Emotional; RP: Role Physical; SC: severe COVID-19 group; SD: standard deviation; SF36v2: Short Form 36 version 2; SF: Social Functioning; VT: Vitality; Δ: change

SF36v2	Severe COVID-19 group (SC) vs mild/healthy (m/h) cases	Mean (SD)	Median (IQR)	P-value (comparison between present and past)	P-value (comparison between SC and m/h)
ΔPF	SC 41–60	-9.41 (12.73)	-5.00 (17.50)	0.004	0.001
	m/h	2.36 (8.58)	0.00 (3.75)	0.128	
	SC 61–80	-7.75 (23.20)	0.00 (18.75)	0.107	0.320
	m/h	-3.13 (14.82)	0.00 (8.75)	0.206	
ΔRP	SC 41–60	-7.35 (23.82)	0.00 (28.13)	0.111	0.029
	m/h	9.72 (22.28)	0.00 (12.50)	0.004	
	SC 61–80	-9.38 (21.89)	0.00 (15.63)	0.024	0.560
	m/h	-3.13 (10.70)	0.00 (10.94)	0.130	
ΔBP	SC 41–60	-13.29 (21.24)	0.00 (33.00)	0.010	0.010
	m/h	3.11 (14.97)	0.00 (0.00)	0.377	
	SC 61–80	0.05 (32.99)	0.00 (15.00)	1.000	0.987
	m/h	3.81 (14.60)	0.00 (0.75)	0.157	
ΔGH	SC 41–60	-9.41 (14.99)	-5.00 (13.50)	0.010	0.000
	m/h	3.86 (14.10)	0.00 (5.00)	0.0545	
	SC 61–80	-5.25 (16.93)	0.00 (10.00)	0.091	0.369
	m/h	-1.25 (12.34)	0.00 (9.75)	0.346	
ΔVT	SC 41–60	-11.76 (20.76)	-5.00 (25.00)	0.037	0.018
	m/h	1.25 (13.17)	0.00 (10.00)	0.277	
	SC 61–80	-7.75 (16.82)	0.00 (18.75)	0.265	0.404
	m/h	-2.50 (7.75)	0.00 (5.00)	0.108	
ΔSF	SC 41–60	8.88 (17.56)	0.00 (13.00)	0.0265	0.009
	m/h	2.81 (13.34)	0.00 (0.00)	0.190	
	SC 61–80	-4.30 (19.11)	0.00 (12.00)	0.546	0.236
	m/h	3.13 (12.50)	0.00 (0.00)	0.167	
ΔRE	SC 41–60	-4.82 (14.13)	0.00 (12.50)	0.217	0.037
	m/h	5.53 (17.40)	0.00 (8.00)	0.0325	
	SC 61–80	-6.70 (21.44)	0.00 (14.75)	0.211	0.560
	m/h	-2.13 (18.94)	0.00 (24.00)	0.918	
ΔMH	SC 41–60	-7.29 (11.51)	0.00 (12.00)	0.0095	0.009
	m/h	2.78 (12.96)	0.00 (7.00)	0.104	
	SC 61–80	-2.80 (8.72)	0.00 (10.00)	0.084	0.028
	m/h	0.75 (7.90)	0.00 (4.00)	0.355	
ΔMHS	SC 41–60	-4.10 (7.89)	-2.01 (6.75)	0.024	0.049
	m/h	1.99 (8.98)	0.00 (4.58)	0.837	
	SC 61–80	-3.06 (6.54)	-1.39 (4.49)	0.025	0.648
	m/h	-0.05 (5.39)	0.00 (6.81)	1.000	
ΔPHS	SC 41–60	-4.24 (6.86)	-2.39 (9.14)	0.0105	0.003
	m/h	1.83 (5.13)	0.45 (2.90)	0.030	
	SC 61–80	-2.45 (10.50)	0.00 (6.27)	0.438	0.262
	m/h	-0.53 (5.29)	0.00 (4.92)	0.347	

**Table 6:** Comparison of EQ5D5L and FACIT-F outcomes between the severe COVID-19 group and mild/healthy (m/h) cases based on age. COVID-19: coronavirus disease 2019; EQ5D5L: EuroQol 5 Dimensions 5 Levels; EQ VAS: EuroQol Visual Analogue Scale; FACIT-F: Functional Assessment of Chronic Illness Therapy-Fatigue; IQR: interquartile range; m/h: mild/healthy; SC: severe COVID-19 group; SD: standard deviation; Δ: change

EQ5D5L	Severe cases (SC) vs mild/healthy (m/h) sample	Mean (SD)	Median (IQR)	P-value (comparison between present and past)	P-value (comparison between SC and m/h)
ΔEQ VAS	SC 41–60	-7.53 (9.01)	-5.00 (11.50)	0.005	0.001
	m/h	2.94 (15.18)	0.00 (0.00)	0.634	
	SC 61–80	-4.00 (14.01)	-2.50 (13.75)	0.156	0.033
	m/h	0.31 (2.21)	0.00 (0.00)	0.290	
ΔIndex Value	SC 41–60	-0.071 (0.144)	0.000 (0.101)	0.036	0.105
	m/h	0.013 (0.132)	0.000 (0.000)	0.638	
	SC 61–80	-0.063 (0.153)	0.041 (0.134)	0.040	0.095
	m/h	-0.037 (0.153)	0.000 (0.000)	0.173	
ΔFACIT-F	SC 41–60	-4.65 (9.67)	0.00 (6.00)	0.0325	0.668
	m/h	0.19 (7.40)	-1.00 (2.00)	0.037	
	SC 61–80	-1.60 (7.26)	0.00 (6.00)	0.180	0.336
	m/h	0.06 (7.58)	0.00 (5.50)	0.487	

Possible correlations were investigated regarding the results of the various questionnaires used in the study (Table 7). Using the Spearman correlation coefficient, a statistically significant positive correlation of variation in FACIT-F with variations of practically all parameters of the SF36v2 and EQ5D5L was detected. Similarly, changes in the Index Value and the EQ VAS correlated positively, at various degrees, with changes in all the SF36v2 parameters.

**Table 7:** Statistical significance of correlations between the four questionnaires’ parameters. Use of Spearman correlation coefficient. BP: Body Pain; EQ5D5L: EuroQol 5 Dimensions 5 Levels; EQ VAS: EuroQol Visual Analogue Scale; FACIT-F: Functional Assessment of Chronic Illness Therapy-Fatigue; GH: General Health; MH: Mental Health; MHS: Mental Health Score; PCFSS: Post-COVID-19 Functional Status Scale; PF: Physical Function; PHS: Physical Health Score; RE: Role Emotional; RP: Role Physical; SF36v2: Short Form 36 version 2; SF: Social Functioning; VT: Vitality; Δ: change

Correlations	ΔFACIT-F (p)	Δ Index Value (p)	ΔEQ VAS (p)	PCFSS (p)
SF36v2 [r(149)]				
ΔPF	0.200 (0.014)	0.297 (0.000)	0.416 (0.000)	-0.370 (0.000)
ΔRP	0.249 (0.002)	0.328 (0.000)	0.228 (0.005)	-0.236 (0.027)
ΔBP	0.240 (0.003)	0.288 (0.000)	0.243 (0.003)	-0.217 (0.043)
ΔGH	0.203 (0.012)	0.198 (0.015)	0.282 (0.000)	-0.242 (0.023)
ΔVT	0.479 (0.000)	0.337 (0.000)	0.265 (0.001)	-0.212 (0.047)
ΔSF	0.318 (0.000)	0.256 (0.002)	0.246 (0.002)	-0.152 (0.159)
ΔRE	0.291 (0.000)	0.330 (0.000)	0.195 (0.016)	-0.165 (0.125)
ΔMH	0.436 (0.000)	0.414 (0.000)	0.309 (0.000)	-0.261 (0.014)
ΔMHS	0.370 (0.000)	0.374 (0.000)	0.214 (0.008)	-0.247 (0.020)
ΔPHS	0.149 (0.068)	0.209 (0.010)	0.228 (0.005)	-0.179 (0.095)
FACIT-F [r(149)]				
ΔFACIT-F	1.000	0.396 (0.000)	0.352 (0.000)	-0.291 (0.006)
EQ5D5L [r(149)]				
ΔIndex Value	0.396 (0.000)	1.000	0.597 (0.000)	-0.455 (0.000)
ΔEQ VAS	0.352 (0.000)	0.597 (0.000)	1.000	-0.511 (0.000)
PCFSS [r(86)]	-0.291 (0.006)	-0.455 (0.000)	-0.511 (0.000)	1.000

Finally, values of PCFSS, the newest scale, correlated negatively with the variation of scores of the other three questionnaires. A statistically significant positive moderate correlation between age and values of the PCFSS was also observed, as was expected.

## Discussion

The COVID-19 pandemic has caused significant socioeconomic and psychological burden for individuals and societies alike [1,13]. This study attempts to perform an analysis of the long-lasting HRQL deterioration exhibited by patients who had severe COVID-19 in Greece. The aforementioned results imply that patients with severe disease requiring hospitalization showed a marked decline in parameters associated with both physical and psychological health. Despite the small sample, this decline was shown to be statistically significant in comparison both to their former clinical status and the performance of patients with mild COVID-19 or healthy individuals. The fact that this drop in health status is detected six months after the disease is further proof of the long-term effects of COVID-19 [1-3]. Patients who needed hospital treatment due to COVID-19 mount in tens of thousands in Greece, making symptomatology due to long COVID-19 a common clinical entity and a problem that needs to be aggressively addressed. The use of any of the above-mentioned questionnaires can help in detecting patients most in need of interventions because there appears to be a significant correlation between the results of the different scores.

Our conclusions seem to be in line with international research regarding the risk factors for post-COVID-19 of the female gender, disease severity, presence of comorbidities, and age [2-4,11,20,24]. Of note is the finding of psychological health decline in those of younger age who had a milder disease or were not infected at all. This could possibly be attributed to the stress associated with prolonged lockdowns and the accompanying mitigation of socioeconomic life.

However, several limitations exist in our study, the foremost of which is the small sample of patients, derived from one hospital, and the uneven distribution of patients between groups. Of note, many formerly hospitalized patients proved difficult to reach or denied participation altogether, while patients younger than 40 and with a history of severe COVID-19, indeed rare, did not take part at all. There was no concomitant clinical and laboratory follow-up of these patients apart from the form-completion visits. Despite these, the role of severe disease in the emergence of post-COVID-19 symptomatology is undeniable, as shown by the study’s results, and therefore, there is an urgent demand for specialized health policy adoption.

We propose the founding of post-COVID-19-centered medical practices, within the scope of already existing hospital facilities, which will serve in detecting and monitoring survivors of COVID-19-associated hospitalization. In addition, rehabilitation and psychological support programs will help reinstitute survivors to their former health status and alleviate the consequences of the disease. Further investigation is needed to determine the optimal mix of multidisciplinary approaches for each type of patient depending on their symptoms. This process would help avoid additional burdens on the social and health services of Greece in the long run because post-COVID-19 syndrome constitutes an entity expected to hinder the healthcare system’s and workforce’s function for years to come.

## Conclusions

Despite the small sample size of our study, the impact of age, gender, and severity of COVID-19 is evident on the possibility of post-COVID-19 symptomatology emergence, as detected by the use of different scales and questionnaires. The wide spread of the virus gives rise to a secondary epidemic among survivors that needs to be addressed under a multidisciplinary scope before it amounts to a disproportionate burden for the Greek national healthcare system. Access of patients to rehabilitation and psychological support services is essential for their complete recuperation and restoration of independent function. Adopting such a policy and keeping the public informed are essential measures to invoke awareness about post-COVID-19 syndrome, as well as to meet the challenge it poses. Allocation of the necessary resources will prove difficult, but ignoring the problem will inevitably lead to far greater expenses in the future.
